# Machine learning–based long-term degradation and LCOE Analysis of floating PV with custom pontoon design

**DOI:** 10.1371/journal.pone.0342926

**Published:** 2026-07-14

**Authors:** Roby Mohajon, Md Rabiul Islam Polash, Md. Nabil Shahriar, Anupom Bhowmick, Pranab Kumar Mondol, Hrittik Mutsuddi, Jihanul Haque Jihan, Nur Mohammad

**Affiliations:** 1 Department of Electrical and Electronic Engineering, Barishal Engineering College, University of Dhaka, North Durgapur, Barishal, Bangladesh; 2 Department of Electrical and Electronic Engineering, Chittagong University of Engineering and Technology, Chittagong, Bangladesh; University of Mazandaran, IRAN, ISLAMIC REPUBLIC OF

## Abstract

Floating Photovoltaic (FPV) systems are a feasible alternative for solar energy utilization in areas where land is scarce. However, issues related to performance degradation, ecological effects, and economic viability have restricted the widespread acceptance of this technology. This study examines the eco-compatibility of a 5 MW FPV solar plant, which is suitable for a wetland ecosystem in Bangladesh, and examines the technical feasibility, environmental sustainability, and economic viability of the plant. In this study, a model was developed using detailed system-level simulations, and the model validated the structural feasibility of a specially designed light-permeable annular pontoon by performing hydrostatic buoyancy and stability tests. Ecological compatibility is assessed using a light-transmission-based photosynthetic viability model, whereas the long-term degradation of the performance ratio and energy yield over a 25-year lifetime is predicted using a climate-aware machine learning framework that incorporates irradiance, temperature, humidity, and system aging effects. Furthermore, an economic model sensitive to inflation was used to examine the levelized cost of electricity (LCOE) in the case study. The results show an average performance ratio of 82.4%, lifecycle energy outputs of approximately 222 GWh, and an LCOE of 0.0315 USD/kWh. In addition, approximately 70% of the aquatic photosynthesis potential is retained, and approximately 104,149.5 tCO₂ is eliminated.

## 1 Introduction

The transition to decarbonized power systems has seen photovoltaics emerge as a key component of national energy policies; however, traditional ground-mounted PV systems exert ever-increasing land pressures in areas characterized by high population densities and agriculture-related land constraints [1,2]. Floating Photovoltaics (FPVs), on the other hand, provide a viable avenue for overcoming these constraints through the use of inland waterbodies, such as lakes and wetlands, as installation platforms, with the added benefits of water cooling of modules and the reflective albedo effect for improved irradiation owing to their biphotonic nature [3,4]. Given a global technical potential of approximately 22TWp for inland reservoirs [5], FPVs are particularly significant for countries such as Bangladesh, which suffers from limited available land, has ample inland water resources, and has set an energy target of 30% renewables by 2030 [6].

Nonetheless, a robust and credible approach to conducting long-term FPV assessments entails acknowledging some challenges associated with methodologies that cannot be addressed by traditional models. Photovoltaic performance deterioration is widely known to be a nonlinear phenomenon affected by climatic factors such as irradiance fatigue, thermal stress, and moisture intrusion. However, traditionally, photovoltaic performance degradation is considered a constant annual value, thus leading to a gradual increase in errors during the forecasting of energy yield in terms of decades [7–10]. In economic terms, the application of a traditional static or gradually increasing value for operating expenses fails to reflect the real impact of inflation on maintenance and replacement costs, particularly in developing countries [11]. Finally, in view of the above, traditional FPV installations are bound to reduce the photosynthetic productivity of water ecosystems [12,13].

This study proposes an eco-techno-economic analysis of a 5 MW Floating Photovoltaic (FPV) setup at Tanguar Haor, Bangladesh. Light-passing annular pontoon designs have been suggested, with preliminary hydrostatic stability considerations followed by a physical analysis of the ecological aspect of the structure using a photosynthesis-irradiance model. Energy efficiency was established by simulations in PVsyst software, while 25-year performance ratio degradation modeling based on climate characteristics and plant age was considered using a Supervised Random Forest Regression (RFR). The unique combination of a light-passing pontoon structure, a degradation model based on machine learning, and cost estimation considering the effects of inflation forms the essence of the current research effort.

## 2 Literature review

### 2.1 FPV system design and hydrostatic stability

The design of floating PV structures necessitates the joint fulfillment of both the electric and hydrodynamic criteria. Early studies on structural analysis proved the feasibility of buoyancy support for inland HDPE flotation rafts based on static loads [14,15]. Nevertheless, such investigations were predominantly based on static assumptions, which may not adequately reflect the complex dynamics of mooring interaction and wave coupling under storm conditions. Hydrodynamic simulations have revealed that the connector forces can be two to three times greater than the static estimates for moderate sea conditions [16], whereas finite element models have shown that the connector joints are vulnerable to fatigue damage owing to repeated waves [17]. Both effects are beyond the scope of the hydrostatic models. There has been relatively little systematic work on the physics of mooring response in shallow water bodies, particularly in highly fluctuating water levels, such as the haor wetlands of Bangladesh. To the best of our knowledge, no existing study has combined a light-transmissive pontoon design with a metacentric stability assessment. These considerations led to the development of the structural module described herein.

### 2.2 Techno-economic assessment of FPV systems

Despite the continued relevance of the levelized cost of energy (LCOE) as the prevailing FPV economic indicator, both the application and calculation of this metric have been compromised by two interrelated simplifications. For instance, most techno-economic evaluations employ an invariant annual degradation rate regardless of climate conditions [18,19], reducing a highly nonlinear phenomenon influenced by humidity and temperature factors to a simple scalar that could overstate the predicted energy output in tropical climates [8]. In addition, the operational expenses (OPEX) were modeled as a constant value or linearly increasing percentage of the capital costs (CAPEX). Such an approach ignores the exponential nature of price increase effects, which often prevail in developing countries. Literature evidence suggests that a deterministic framework that considers degradation and LCOE adjustment can significantly alter cost projections compared to a constant-output scenario [20]. However, to the best of our knowledge, no previous study has incorporated climate-based degradation and OPEX escalation rates in FPV projects in Bangladesh, where inflation rates of 6–12% are well documented.

### 2.3 Machine learning for PV performance modeling

The integration of machine learning (ML) models for PV system performance analysis is on the rise; however, architectural considerations are seldom informed by the restrictions imposed by the dataset structures. ANN models are prone to initialization issues and overfitting when used on small training sets [21]. While LSTMs are theoretically well-suited for analyzing long sequences of data, they require large sets of field measurement data, which would not be readily available for future site assessments in data-poor developing countries [22]. XGBoost regression models are considered robust, but their performance can potentially benefit from extensive hyperparameter tuning in proportion to the amount of data available [23]. In contrast, RFR offers several advantages: it does not suffer from overfitting owing to ensemble averaging, allows for efficient model computations, has minimal hyperparameter sensitivity, and provides a built-in permutation approach for interpreting input features [24]. Climate-dependent RFR models have proven to be superior to the assumption of a fixed degradation rate when applied to large commercial PV monitoring data [7]. Nevertheless, incorporating RFR-based degradation modeling into multi-decadal FPV system techno-economic assessments remains understudied. Prior studies on ML techniques in the FPV domain have focused primarily on short-term performance prediction and fault detection applications.

### 2.4 Environmental and ecological considerations

FPV panels limit the availability of photosynthetically active radiation (PAR), with possible ecological implications depending on the absolute levels of PAR under the subarray and not solely on the percent shading. From the kinetic Michaelis-Menten model, PAR below 15 W m ⁻ ² appears to correlate with the onset of heterotrophy in some aquatic systems [25]. However, whether this is a universal threshold value for wetland typologies remains unknown. Laboratory simulations of FPV applications in a UK reservoir observed significant phytoplankton biomass inhibition with an increased FPV area; however, such results are not necessarily relevant in tropical wetlands, given that the mixing regime induced by monsoons and ambient temperatures differs from those of temperate regions [26]. In Germany, surface water temperatures were reduced by 1–3°C under FPVs, and below a coverage ratio of 40%, minimal microalgae growth was found to occur in these specific reservoirs [27]. These observations are geographically specific and applicable to other environments only within reason. Biodiversity monitoring in FPV research has been largely heterogeneous. The most commonly adopted design solution, in which the coverage ratio is limited to 30%−40% [28], is generic to all sites and does not consider whether it would be possible to optimize the pontoon geometry to enhance the transmission of light.

### 2.5 Research gap and motivation

Four interrelated gaps arise from this review. To the best of the authors’ knowledge, no formal metacentric height analysis of the hydrostatic stability of light-permeable and annular pontoon designs has been reported in the literature. From an economic standpoint, techno-economic analyses of FPV systems that account for inflation-driven OPEX increases under degradation driven by climate conditions are still relatively underexplored, especially in Bangladesh. In computational terms, the use of RFR-based supervised machine learning for the long-term techno-economic assessment of FPVs remains unexplored, considering the role of climate. Ecologically, the coupling of structural design for light permeability with a physical model for photosynthetic viability is underdeveloped. These gaps are addressed in the current study via (i) metacentric height and buoyancy safety factor analysis of the novel annular pontoon design; (ii) PVsyst climate simulations at Tanguar Haor based on NASA POWER reanalysis; (iii) long-term RFR-based 25-year performance ratio and degradation analysis under the influence of climate-induced features; (iv) inflation-driven, degradation-accounting LCOE analysis under ADB/IMF macroeconomic assumptions; and (v) a Michaelis–Menten photosynthetic viability analysis of the proposed pontoon design.

## 3 Methodology

### 3.1 Site geographical and elevation profile of the study area

The suggested FPV system will be established in Tanguar Haor, an area in the Sunamganj District located in northeastern Bangladesh. The selection of this particular location was performed after considering factors such as hydrological appropriateness, land restrictions, ecological sensitivity, and renewable potential. The Sunamganj District contains many haors that are flooded throughout the year. Therefore, the utilization of FPV in this area would not conflict with agricultural and urban land uses. According to the digital elevation analysis based on the open-source SRTM DEM data, the landscape in this study area is relatively flat, with elevations less than 10 m above the MSL. Consequently, FPV installations in low-lying terrains do not involve major engineering work. Tanguar Haor, a Ramsar site, has a massive body of open water, low levels of navigation, and less infrastructure. Thus, Tanguar Haor is one of the best locations for modular floating PV systems. Eco-sensitivity was assessed through the adoption of an FPV design, where floating elements allow the passage of sunlight, thus reducing ecological damage. As shown in Table 1, the chosen site has an excellent balance of solar energy availability and hydrological attributes. With respect to solar irradiance, there is a notable presence of global and diffuse radiation, which makes it well-suited for the use of bifacial PV modules under wet conditions. Hydrologically, the relatively high surface water area and shallow depths (2–6 m) allow for easy installation while ensuring anchorage and mooring stability.

**Table 1:** Site characteristics and solar resource parameters of Tanguar Haor [29].

Particulars	Descriptions
**Project site**	Tanguar Haor
**Daily global solar irradiance**	4.4–4.8 kWh/m²/day
**Daily diffuse solar irradiance**	1.9–2.3 kWh/m²/day
**Annual global solar irradiance**	1,600–1,750 kWh/m²/year
**Annual diffuse solar irradiance**	700–850 kWh/m²/year
**Total water surface area**	9,700 ha
**Depth**	2-6 m
**Maximum depth**	8-10 m

### 3.2 Solar resource data analysis

**Fig 1:**
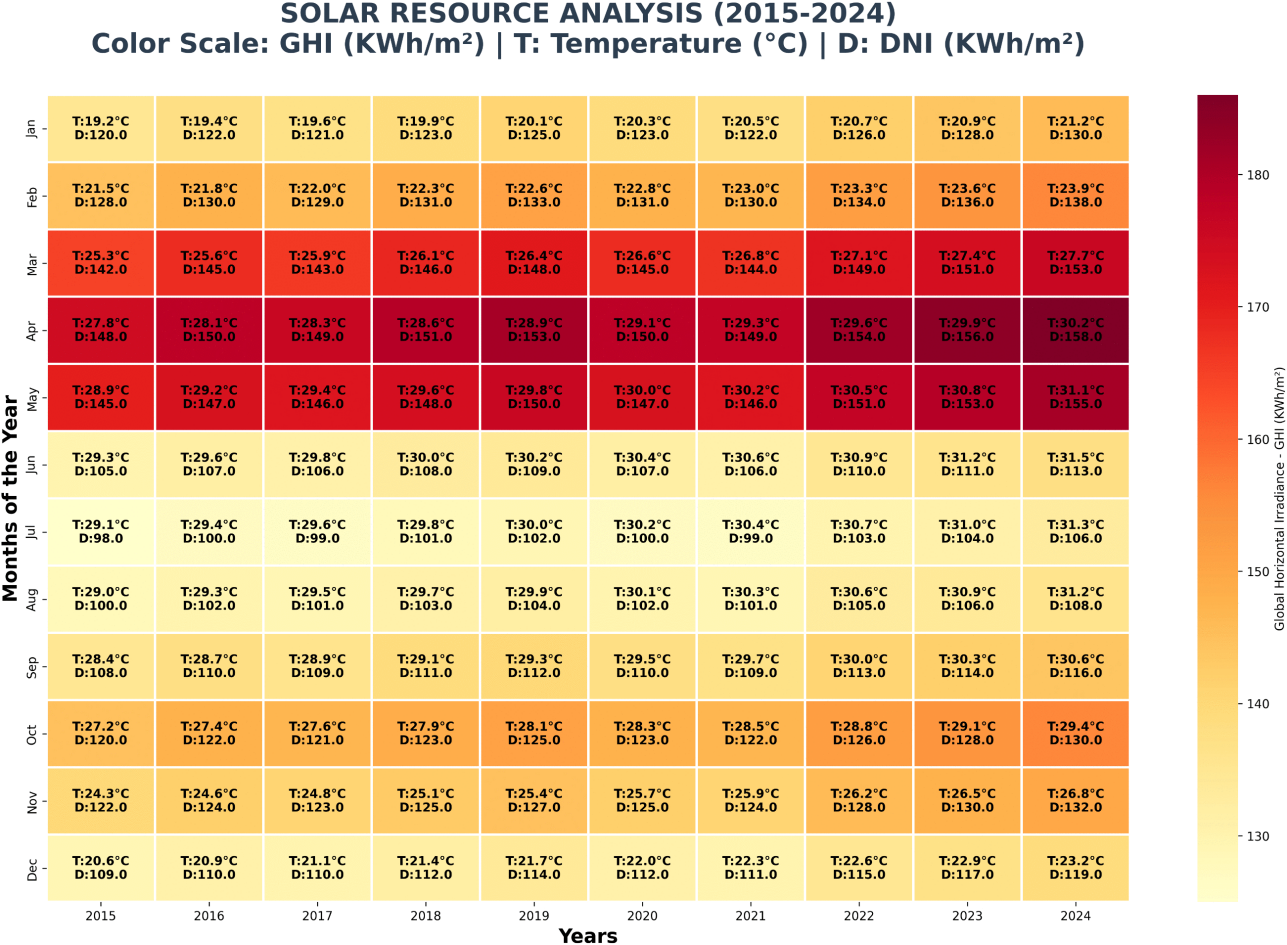
Monthly solar resource data visualization (2015–2024).

Fig 1 provides a holistic representation of the long-term availability of the solar resource at the study site from 2015–2024 [30]. The graph combines the monthly values of the Global Horizontal Irradiance (GHI) resource and provides heat maps, where the relevant annotations include the mean air temperatures and values of the Direct Normal Irradiance (DNI). These graphical representations facilitate the joint analysis of both radiative and thermal resource parameters, which play a significant role in defining the performance and degradation of floating photovoltaic facilities. These factors indicate the existence of distinct intra-annual cycles of radiative resource parameters, where the GHI and DNI fluxes reach their peak values during the pre-monsoon periods of March, April, and May, influenced by high solar heights and lower values of clouds, sky obstructions, and turbidity. In contrast, the monsoon period of June, July, and August revealed a distinct depletion of radiative resource parameters, primarily influenced by the high values of clouds and sky obstructions and high water content of the atmospheric column, while the post-monsoon periods of September, October, and November revealed moderate resource values, accompanied by reduced temperatures, mostly favorable conditions from the perspective of photovoltaic cell efficiency, primarily influenced by reduced cell surface temperatures. The yearly variations from to 2015–2024 reveal relatively stable conditions of radiative resource parameters and no extreme variations, thus facilitating the reliability of the region’s resource parameters from the perspective of accurate long-term energy output predictions and allocations. The thermal conditions indicate distinct alterations and increments in the mean air temperatures during the investigation period, which is particularly relevant when analyzing floating photovoltaic setup parameters, primarily influenced by reduced cell surface temperatures. The combined radiative and thermal analysis clearly shows that the study location has high inter annual variability but low uncertainty; therefore, this is a good foundation for further machine learning-based performance degradation analysis.

### 3.3 System design and key component specifications

The floating solar photovoltaic (FPV) project would consist of 9,000 bifacial monocrystalline PV modules with a capacity of 700 Wₚ. Therefore, the total installed DC power is 6.3 MWₚ. The modules were arranged in a string of 500 strings with 18 modules in series in each string. This ensures electrical compatibility with the selected inverter configuration. The DC connection was made with the aid of 50 units of Huawei SUN2000–100KTL-M1 three-phase 480V grid-connected string inverters with an overall capacity of 100 kW AC each. Consequently, the total installed AC capacity was 5 MW with a DC/AC ratio of 1.26. The crucial electrical, thermal, and dimensional parameters of the PV modules and inverters are presented in Table 2. Simulations of energy production were carried out using PVsyst software with typical climatic data, utilizing the meteorological data typical of the Tanguar Haor area provided by Meteonorm 8.1 software. The conditions used include global horizontal irradiance between 1,900−2,000 kWh/m^2^/year, an average ambient temperature of 27 °C, and typical wind circulation patterns. The fixed tilt angle of the PV module was chosen to be 12°, which considered the structural stability and floating platform requirements, along with energy production. The loss modeling considered the major losses in the system, namely the mismatch losses of the modules at 2%, DC cable losses of 1.5%, incident angle modifier (IAM) losses of 2.4%, and a thermal loss coefficient of 29 W/$m^{\text{2}}$.K [31].

**Table 2:** Technical specifications of the photovoltaic module and inverter.

Parameter	PV Module (Trina Solar 700 Wₚ)	Inverter (Huawei 100 kW)
**Number of cells**	132	
**Rated power**	700 W	100 kW (Output AC)
**Maximum power / input power**	700 W ($P_{max})$	126 kW (DC input)
**Open-circuit voltage**	48.60 V ($V_{oc})$	1100 V (max DC)
**Voltage at maximum power**	40.5 V ($V_{mpp})$	720 V (rated DC)
**Short-circuit current**	18.32 A ($I_{sc})$	
**Current at maximum power**	17.29 A ($I_{mpp})$	133.7 A (Output AC)
**Efficiency**	22.5%	98.6%
**MPPT voltage range**		200-1000 V
**Number of MPPTs**		10
**Dimensions**	2384 × 1303 × 33 mm	1,035 × 700 × 365 mm
**Operating temperature range**	−40°C to 85 °C	−25°C to 60°C
**Weight**	38.3 kg	93 g

For a PV module with a bifacial structure, a water surface albedo value of between 0.08 and 0.12 was considered to account for the reflected irradiance on the water surface of the reservoir [32]. The positive contribution of this bifacial structure, combined with the passive cooling effect of the water reservoir, improves the performance ratio and overall energy production of PV modules. The system configuration was defined to provide a sound foundation for the performance analysis.

### 3.4 Hydrostatic design and stability analysis of the floating pontoon system

**Fig 2:**
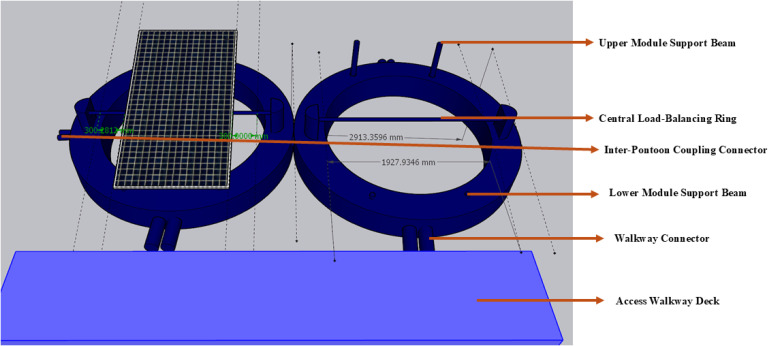
Unit-level geometry of the annular floating pontoon with bifacial PV module.

**Fig 3:**
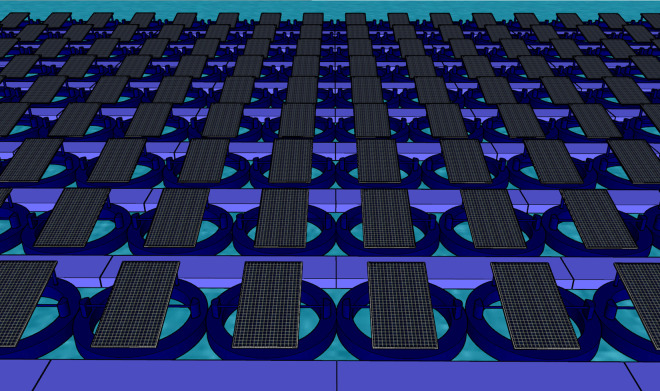
Modular array deployment of the pontoon-based floating PV system.


The hydrostatic feasibility of a proposed floating solar photovoltaic (FPV) design is examined on the basis of geometric arrangements of a unit composed of a pontoon and a photovoltaic module, as shown in Figs 2 and 3. The conceptual design of a unit, which is hydrostatically feasible, comprises an annular pontoon and a bifacial photovoltaic module. At the unit level (Fig 2), the pontoon is designed as a hollow annular structure with an outer diameter $D_{o}=\text{2}.\text{9}\text{1}\text{3}\;\text{m}$ and an inner diameter $D_{i}=\text{1}.\text{9}\text{2}\text{8}\;\text{m}$. Under static equilibrium, the effective submerged height is assumed to be h=0.30m. The analysis considers freshwater conditions with density $\rho =\text{1}\text{0}\text{0}\text{0}\;\text{kg}\;{\text{m}}^{-\text{3}}$ and gravitational acceleration $g=\text{9}.\text{8}\text{1}\;\text{m}\;{\text{s}}^{-\text{2}}$. The displaced water volume of the pontoon is expressed as [33],

$$\begin{array}{c} V=\;\frac{\pi }{\text{4}}\left(\overset{\text{2}}{\underset{O}{D}}-\overset{\text{2}}{\underset{i}{D}}\right).h \end{array}$$

Substituting the geometric parameters yields a displacement volume of approximately $V=\text{1}.\text{1}\text{2}\;{\text{m}}^{\text{3}}$. According to Archimedes’ principle, the resulting buoyant force is given by,


$$\begin{array}{c} F_{b}=\rho gV\;\approx \text{1}\text{0}.\text{9}\text{8}\;KN \end{array}$$

which corresponds to a maximum buoyancy-supported mass of,


$$\begin{array}{c} W_{max}=\;\frac{F_{b}}{g}=\text{1}\text{1}\text{2}\text{0}\;kg \end{array}$$

The applied static load on each pontoon consists of the bifacial photovoltaic module mass $W_{\text{PV}}=\text{3}\text{8}.\text{3}\;\text{kg}$, the pontoon self-weight $W_{\text{p}}\approx \text{6}\text{7}\;\text{kg}$ (assuming high-density polyethylene construction), and the mounting and fastening components $W_{\text{m}}\approx \text{1}\text{5}\;\text{kg}$. The total applied load is therefore,


$$\begin{array}{c} W_{total}=\;W_{pv}+W_{p}+\;W_{m}=\text{1}\text{2}\text{0}\;kg \end{array}$$

The factor of safety against buoyancy failure is defined as [34],


$$\begin{array}{c} {FOS}_{buoyancy}=\;\frac{W_{max}}{W_{total}}=\text{9}.\text{3}\text{3} \end{array}$$

indicating that less than 12% of the available buoyant capacity is utilized under static operating conditions.

Static hydrostatic stability is evaluated using the metacentric height criterion. The second moment of area of the waterplane for the annular pontoon is given by [35],


$$\begin{array}{c} I=\;\frac{\pi }{\text{6}\text{4}}\;\left(\overset{\text{4}}{\underset{O}{D}}-\overset{\text{4}}{\underset{i}{D}}\right) \end{array}$$

which yields $I\approx \text{3}.\text{3}\text{4}\;{\text{m}}^{\text{4}}$. The metacentric radius is then calculated as [36],


$$\begin{array}{c} BM=\frac{I}{V}=\text{2}.\text{9}\text{8}\;m \end{array}$$

Assuming a conservative vertical separation between the center of buoyancy and the center of gravity of the pontoon–module system of BG≈0.40m, the metacentric height becomes [37],


$$\begin{array}{c} GM=BM-BG=\text{2}.\text{5}\text{8}\;m \end{array}$$

Since GM>0 and significantly larger than zero, the pontoon exhibits strong positive static stability and a substantial restoring moment against small angular perturbations.

From the system perspective (Fig 3), several pontoon-module assembly units are placed in a repetitive modular system and connected via longitudinally and transversely aligned flotation beams to create an extensive FPV module system. This design allows for efficient load distribution while ensuring geometric and hydrostatic stability. An equally spaced modular configuration allows for the minimization of module shading while providing partial exposure to the water surface for bifacial energy generation and heat dissipation. The modular arrangement makes it easier to scale up FPV system installation at the utility level. Furthermore, a distributed structural configuration may decrease the formation of stress concentrations owing to hydrodynamic interactions. Nonetheless, this study mainly focused on static hydrostatic load analysis without considering hydrodynamic or CFD modeling under climatic extremities such as hurricane winds, storm surges, and high monsoon waves. Future work must include comprehensive extreme weather simulation tests along with mooring stability tests to evaluate the overall performance in extreme environments.

The general analysis that entails the mathematical representation in geometry, the analysis of buoyancy force capability, and the hydrostatic stability test revealed that the proposed ring-shaped pontoons satisfy all requirements of having adequate carrying capacity, factor of safety, and static stability, thus forming the proper foundation for the floating solar PV system.

### 3.5 Light transmission through side gaps and photosynthetic viability

**Fig 4:**
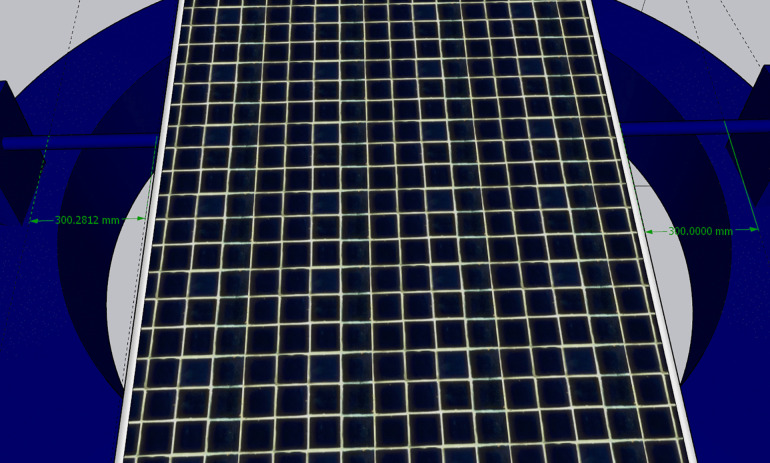
Side-gap geometry of the floating PV module illustrating solar light transmission to the water surface.

As shown in Fig 4, each PV module was placed upon an annular floating pontoon having a lateral gap width of about 300 mm on either side, designed to allow partial solar irradiation to pass through beneath the PV array. A calculation regarding whether this can provide a level of solar irradiation sufficient to facilitate photosynthesis among aqueous organisms was performed using a photosynthesis transfer assessment.

Let the total incident horizontal solar irradiance be denoted by G, which can be decomposed into direct beam ($G_{b})\;$ and diffuse components ${(G}_{d})$ as [38],


$$\begin{array}{c} G=\;G_{b}+\;G_{d} \end{array}$$

The geometric openness of the pontoon–module configuration is defined as [39],


$$\begin{array}{c} f_{o}=\;\frac{\text{2}g}{W_{PV}+\text{2}g} \end{array}$$

where g=0.30m represents the side gap width and $W_{PV}=\text{1}.\text{3}\text{0}\;\text{m }$ is the effective module width. Substituting these values yields $f_{o}=\text{0}.\text{3}\text{1}\text{6}$, indicating that approximately 31.6% of the horizontal footprint remains directly exposed through the side gaps.

The irradiance transmitted beneath the floating array, accounting for geometric openness and diffuse sky contribution, can be expressed as [40],


$$\begin{array}{c} G_{t}=\;f_{o}\;G_{b}+\tau_{d}G_{d}+\;\emptyset_{s}G_{b} \end{array}$$

where $\tau_{d}$ denotes the diffuse sky view factor and $\phi_{s}$ represents edge-scattered radiation from module and pontoon boundaries. Conservatively neglecting edge scattering ($\phi_{s}\to \text{0}$) and adopting $\tau_{d}=\text{0}.\text{7}$, the expression reduces to,


$$\begin{array}{c} G_{t}\;\approx \;f_{o}\;G_{b}+\text{0}.\text{7}\;G_{d} \end{array}$$

Introducing the diffuse fraction $k_{d}=G_{d}/G$, the transmitted irradiance may be rewritten as [41],


$$\begin{array}{c} G_{t}=\;\left[f_{o}\;\left(\text{1}-k_{d}\right)+\text{0}.\text{7}\;k_{d}\right]G \end{array}$$

For typical tropical FPV conditions where $k_{d}\approx \text{0}.\text{4}\text{0}$, the resulting transmissivity becomes $G_{t}=\text{0}.\text{4}\text{6}G$, implying that nearly 46% of ambient horizontal irradiance is available beneath the array.

Photosynthetically active radiation (PAR), which drives aquatic primary production, is obtained from broadband irradiance using [42],


$$\begin{array}{c} PAR=\;\eta_{PAR}\times \;G_{t} \end{array}$$

The parameter $\eta_{\text{PAR}}\approx \text{0}.\text{4}\text{5}\;$ represents the fraction of incoming solar radiation that lies within the photosynthetically active radiation (PAR) waveband of 400–700 nm. Considering the partial shading effect imposed by the floating photovoltaic (FPV) structure, the available PAR beneath the system can therefore be approximated as ${\text{PAR}}_{\text{avail}}=\text{0}.\text{2}\text{1}G$. For a representative daily mean global irradiance of $G=\text{2}\text{1}\text{7}\;\text{W}\;{\text{m}}^{-\text{2}}$, the corresponding available PAR is estimated to be approximately $\text{4}\text{5}\;\text{W}\;{\text{m}}^{-\text{2}}$. The adequacy of this irradiance level to support photosynthetic activity is subsequently assessed using a Michaelis–Menten type photosynthesis–irradiance relationship [43],


$$\begin{array}{c} P(I)=\;P_{max}\;\frac{I}{K_{I}+\text{I}} \end{array}$$

Here, $P_{\text{max}\;}$ denotes the maximum photosynthetic rate, while $K_{I}$ represents the half-saturation irradiance constant. For aquatic macrophytes and phytoplankton, $K_{I}$ is typically reported in the range of 15–25 W m^-2^. Substituting the estimated available irradiance beneath the floating PV system ($I={\text{PAR}}_{\text{avail}}=\text{4}\text{5}\;\text{W}\;{\text{m}}^{-\text{2}}$) and a representative value of $K_{I}=\text{2}\text{0}\;\text{W}\;{\text{m}}^{-\text{2}}\;$ into the photosynthesis–irradiance relationship yields $P(I)/P_{\text{max}}\approx \text{0}.\text{6}\text{9}$. This result indicates that nearly 70% of the maximum photosynthetic capacity can be maintained under the floating PV array, suggesting favorable conditions for sustained biological productivity.

It is important to mention that the amount of predicted PAR underneath the proposed floating structure exceeds the minimum required intensity of 10–20 W/m^2^ covered in the literature for aquatic vegetation. Thus, the intensity of light obtained was also within the required range. Therefore, adding side gaps of size 300 mm, as shown in Fig 4, promotes bio-compatibility. It is also important to mention that instead of conducting an ecological assessment study of the proposed structure, this approach offers qualitative results that provide physics-based insights into its viability.

### 3.6 Integrated system modeling and machine learning workflow

**Fig 5:**
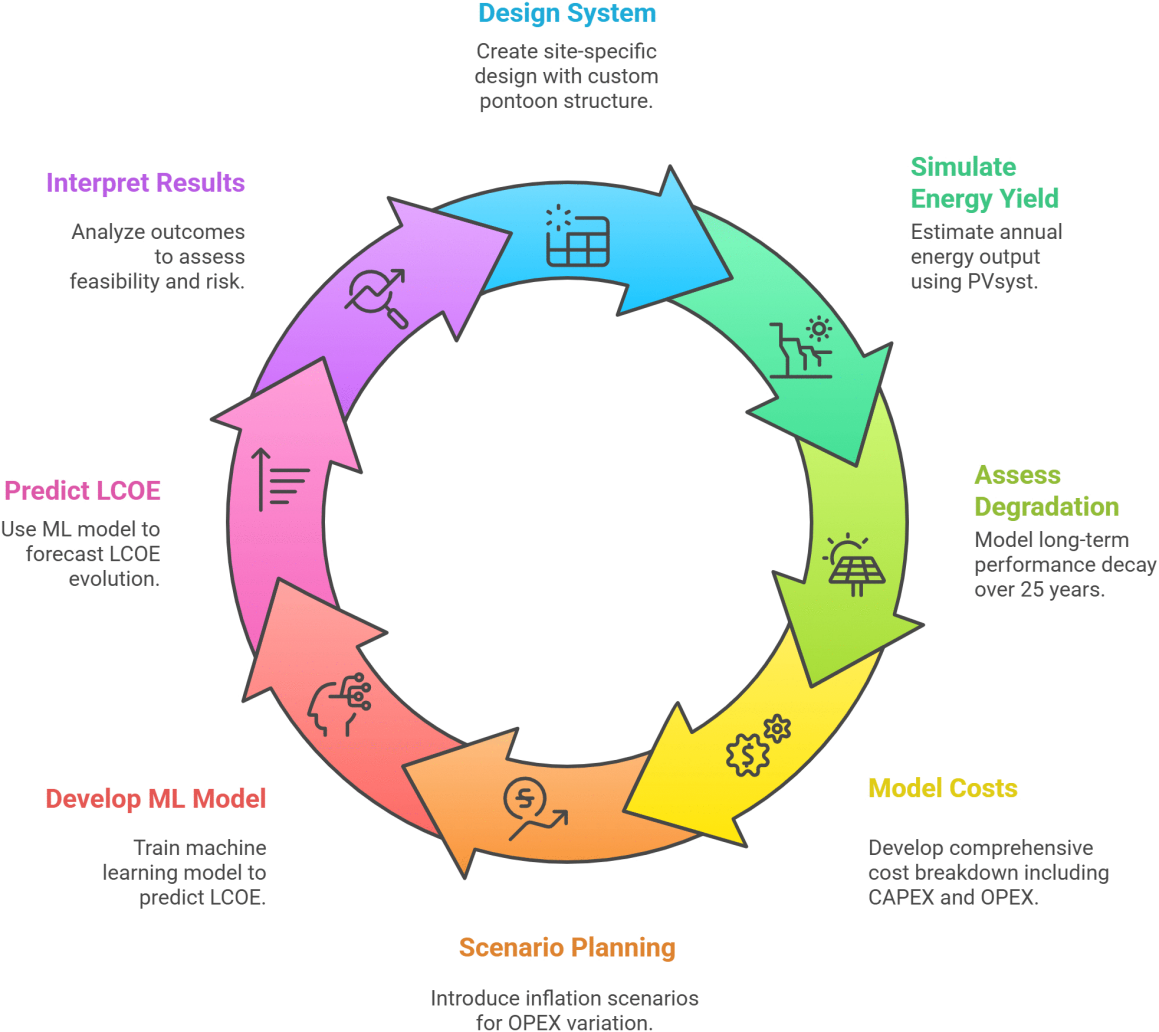
Overall workflow for system design and techno-economic assessment of the floating photovoltaic system.

The general methodology used in this study is shown in Fig 5. The methodology structure was designed to analyze the techno-economic and environmental feasibility of a proposed floating photovoltaic (FPV) power plant with a customized annular pontoon design in terms of its sustainability. This process starts with a regional FPV power plant design phase, considering the hydrologic and climatic features of Tanguar Haor. This helps ensure that the proposed FPV power plant design satisfies both stability and efficiency requirements based on regional environmental factors. An energy generation simulation analysis for the entire life cycle was conducted using the PVsyst tool by incorporating the influence of climatic losses, thermal impacts, and degradation-sensitive operation scenarios to determine the yearly system efficiency. Simultaneously, a techno-economic analysis methodology was designed, which considers both CAPEX and OPEX while focusing on operational cost increase due to inflation and LCOE increase due to degradation.

Supervised machine learning (SML) based framework to predict the techno-economic performance of FPV systems under scenarios throughout their full lifecycle was designed according to the methodology demonstrated in Fig 4. It should be noted that the framework presented in this study is designed as an analysis-informed physical model rather than a predictive tool for FPV performance evaluation fully validated under field conditions. The dataset applied in this research was created using PVsyst climate-aware simulations based on annual energy production, performance ratio decay, CAPEX/OPEX, inflation rate, and FPV system lifetime. Moreover, as far as machine learning algorithms are concerned, only SML regression-based technique was applied in this research using Random Forest Regressor (RFR) approach. Reinforcement learning, coupling of optimization approaches, and multi-agent learning were not considered for FPV performance predictions within the scope of this study.

The first step involved performing a Pearson correlation analysis prior to training the model to evaluate the relationship among the climatic, technical, and economic inputs and the system performance metrics. It was found that global horizontal irradiance had a high positive correlation with yearly energy production, whereas temperature and humidity showed moderate negative correlations with the performance ratio owing to degradation processes. Wind speed had a low positive effect on system performance owing to convective cooling, whereas system age had a negative correlation with system performance. This indicates that the selected input factors are physically significant for the machine learning model.

As part of climate-awareness integration in the degradation model, several climatic parameters, such as GHI, T, RH, and WS, and some operationally related features, such as operational year, annual energy generation, CAPEX, OPEX, inflation rate, and discount rate, were added to the machine learning-based approach owing to their significant impact on the long-term FPV behavior. To investigate the influence of different inputs on the long-term behavior of FPV, a feature importance analysis was conducted using the random forest regression model. The annual energy generation and operational year had the highest influence on the long-term degradation-aware FPV life cycle cost development owing to their inherent role in representing aging and performance degradation during the entire system operation period. The inflation-driven OPEX parameter was identified as another important contributor to the FPV LCOE behavior owing to the long-term effects of its escalation over the years. Regarding climatic factors, T and RH showed a relatively high influence since they contribute to thermal degradation and moisture-induced damage, while wind speed had the least influence since it relates to passive cooling.

Random Forest Regression (RFR) was chosen because of its ability to capture non-linear interactions between the climatic, technical, and economic factors influencing FPV system dynamics. In contrast to ANN and LSTM algorithms, which typically require large field datasets with many features, the current study is based on structured medium-sized datasets produced via PVsyst simulations. In these circumstances, tree-based ensemble approaches exhibit better generalization skills and a lower propensity to overfit. In addition, compared to boosting algorithms such as XGBoost, RFR is characterized by a lower dependence of model performance on hyperparameter tuning for medium-sized structured datasets.

**Fig 6:**
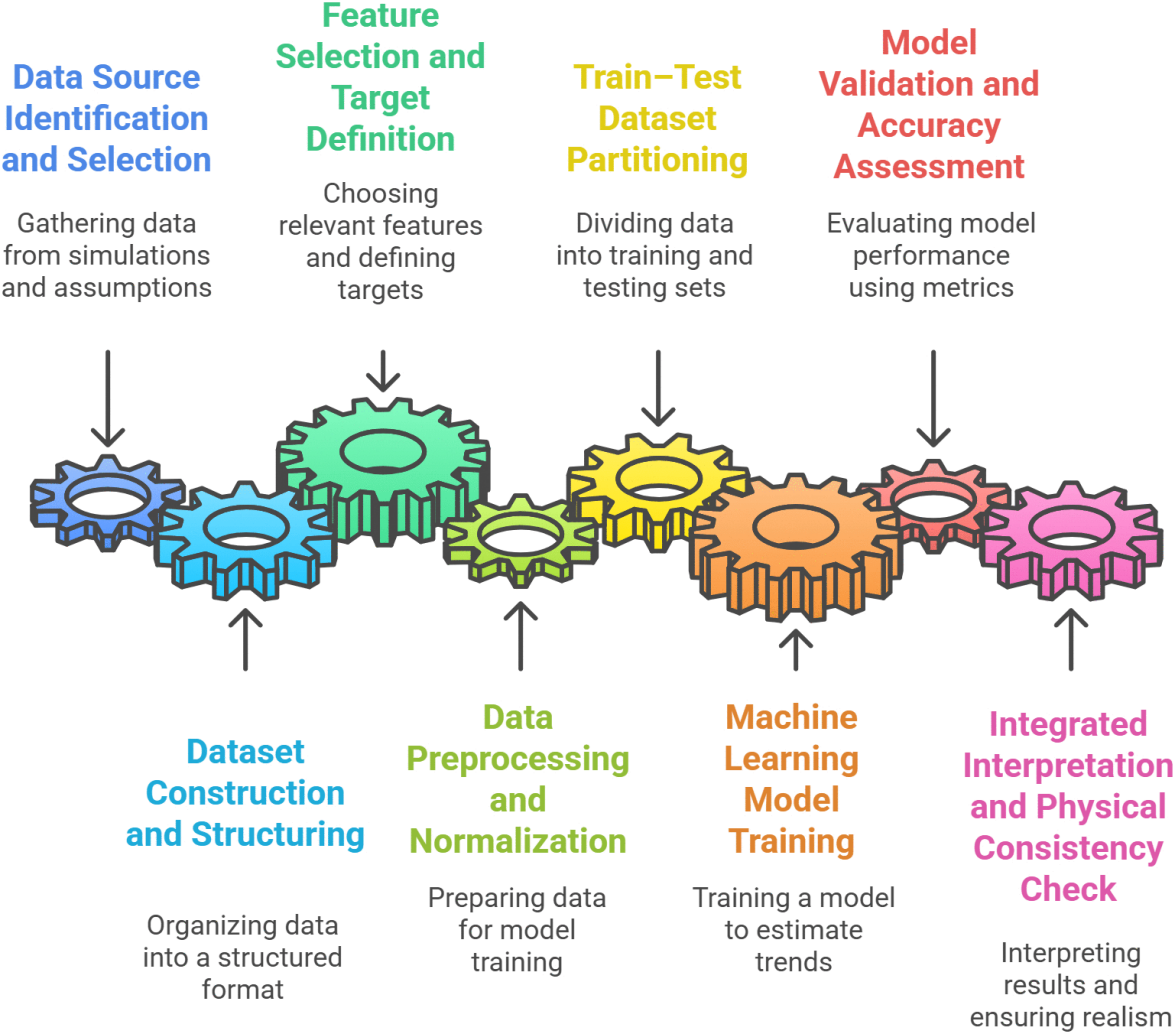
Machine learning framework for predictive modeling and performance evaluation.

The random forest regression model was trained using 300 decision trees with a minimum leaf size of five to avoid overfitting problems. Normalization was performed for all input parameters before training to make the calculations numerically stable and allow equal contribution of all features. The data were split into training and testing datasets at an 80/20 ratio. The performance of the model was assessed using metrics such as the coefficient of determination (R²), root mean squared error (RMSE), and mean absolute error (MAE), and by comparing model predictions with PVsyst techno-economic trajectories (Figs 5, 6).

To assess the strength of the proposed framework, a statistical test for error metrics was conducted. The findings of this test indicate consistent performance through stable R², RMSE, and MAE metrics for the training and testing datasets; hence, the absence of overfitting. Moreover, the predicted degradation trend and LCOE curve-maintained consistency within the typical range of PV degradation (0.6%−1.2% per year), which proves the reliability of the proposed approach in terms of physics-based reasoning.

Although the current study used a simplistic FPV design for illustration, the developed framework is not limited to any particular FPV structure. This is because the methodology does not rely heavily on geometry-related input variables but rather on the climate and economic conditions of the overall system. Consequently, the proposed methodology can be applied to other FPVs with different designs and configurations, such as modular FPV structures with multiple pontoon designs and geometries. Other issues, such as hydrodynamics, temperature effects, and environmental factors, should be considered in future studies.

## 4 Results and discussion

### 4.1 Daily energy yield characteristics of the floating photovoltaic system

**Fig 7:**
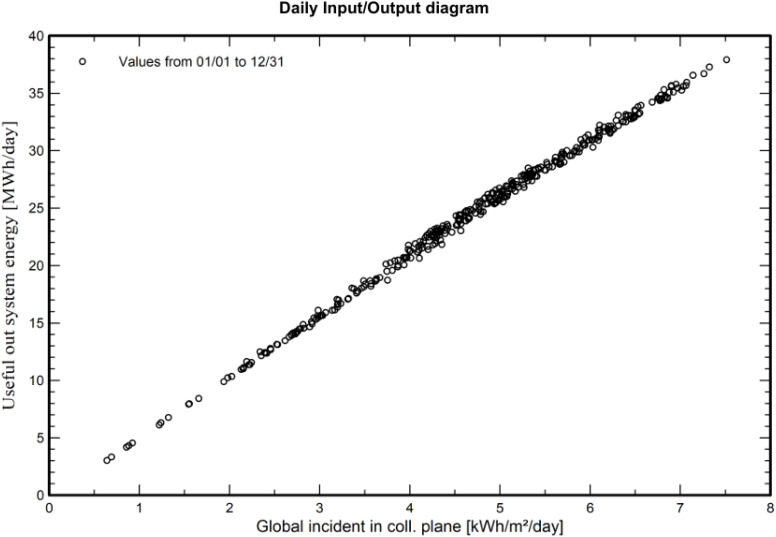
Daily plane-of-array irradiance versus system energy output.

**Fig 8:**
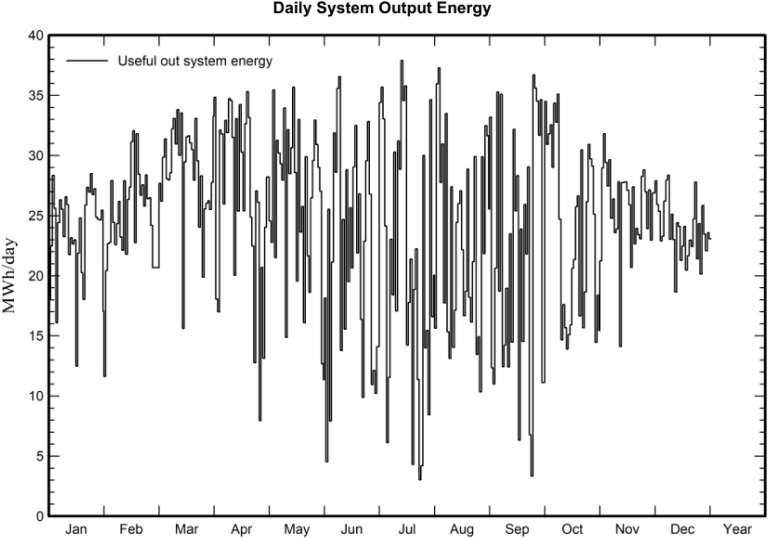
Daily energy output variation of the floating photovoltaic system.

Fig 7 shows the daily relationship between the plane-of-array irradiance and the corresponding system output energy for an entire operational year. The relationship between the plane-of-array irradiance and the daily system output energy is linear, which means that the floating photovoltaic (FPV) system is designed to operate within a stable conversion domain over a wide range of irradiance values. The tight grouping of points along the regression curve depends on the stability of the system performance and the absence of nonlinear losses over a low-irradiance domain, which is indicative of the control over the electrical, thermal, and mismatch losses of the system. Fig 8 highlights the monthly changes in the system output energy for each day of the year. Large day-to-day changes in system outputs can be noted, which are mainly dependent on short-term changes in solar intensity due to cloud cover and weather. Increased daily outputs were mostly recorded in the latter part of spring and summer, whereas decreased and random outputs were noted in the monsoon and winter. However, the system always remains equipped with a higher output margin, irrespective of the cloud cover intensity. The combined interpretation of Figs 7 and 8 focuses on the importance of both high-irradiance responsivity and functional resilience of the FPV model. The linear function between irradiance values and energy demonstrates appropriate model functionalities with predictability suitable for modeling and forecasting, despite the realistic daily output pattern based on appropriate meteorological effects rather than model inefficiency. The obtained correct results can serve as a proper basis for modeling and machine learning predictions related to performance ratio estimations in subsequent sections.

### 4.2 Module temperature behavior and array power output characteristics

**Fig 9:**
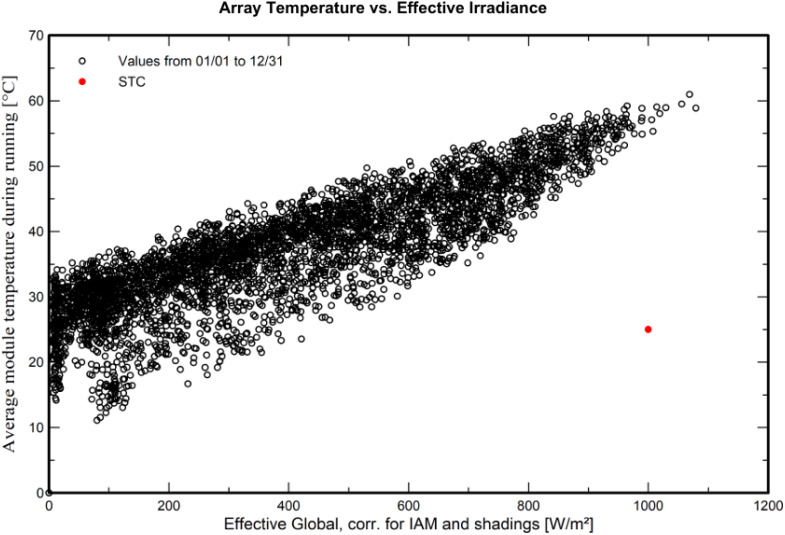
Relationship between effective plane-of-array irradiance and average photovoltaic module operating temperature.

Fig 9 demonstrates the correlation between the effective plane-of-array irradiance corrected for the Incidence Angle Modifier (IAM) and shading impacts and the average operating temperature of photovoltaic modules in a given year. A positive correlation exists between these two parameters, indicating that increasing irradiance levels are accompanied by a corresponding rise in module temperatures due to thermo-electrical coupling in a solar cabinet system, as increased photon absorption translates into elevated semiconductor junction temperatures due to increased absorbency, irrespective of the Standard Test Conditions (STC). The data points are concentrated in a temperature domain that is largely higher than that corresponding to the Standard Test Conditions (STC). This indicates that there are losses that depend on temperature considerations in a system that must be accounted for in a model representation that considers the impact of solar irradiation on a solar panel system under varying irradiation conditions. The data dispersion in Fig 8 can be assumed to be due to variations in transient meteorological conditions. In a situation where a solar panel system has been placed in a floating solar environment, this dispersion indicates that there are conditions under water that work towards reducing increased high irradiation conditions owing to evaporative cooling mechanisms that tend to reduce the increase in semiconductor junction temperature in a solar panel system under irradiation conditions.

**Fig 10:**
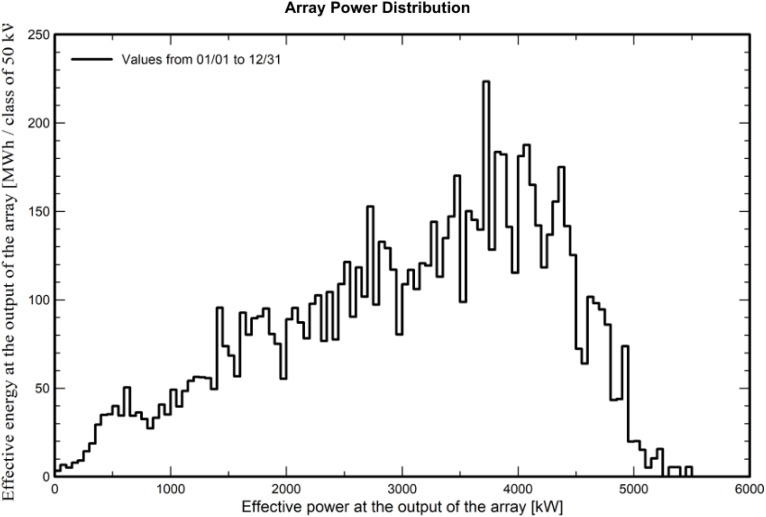
Annual distribution of effective array power output of the floating photovoltaic system.

Fig 10 shows a graphical representation of the distribution of the effective array power output for the same year. The distribution clearly reveals that the array mostly operated at intermediate to high power levels, and most of its operating hours were concentrated around the higher end of the distribution. The lower end of the distribution is mainly concentrated around early morning, late afternoon, and cloudy conditions, whereas the cut-off at the higher end is due to inverter clipping at higher irradiations.

The joint analysis is shown in Figs 9 and 10 clearly reveal that while higher irradiations cause higher panel temperatures, the power output distribution is still optimal and satisfactory for the year, as higher irradiations do not pose any problems to the floating PV layout, thereby forming a good platform for the analysis of performance ratio and degradation.

### 4.3 Monthly energy conversion performance of the floating PV system

**Fig 11:**
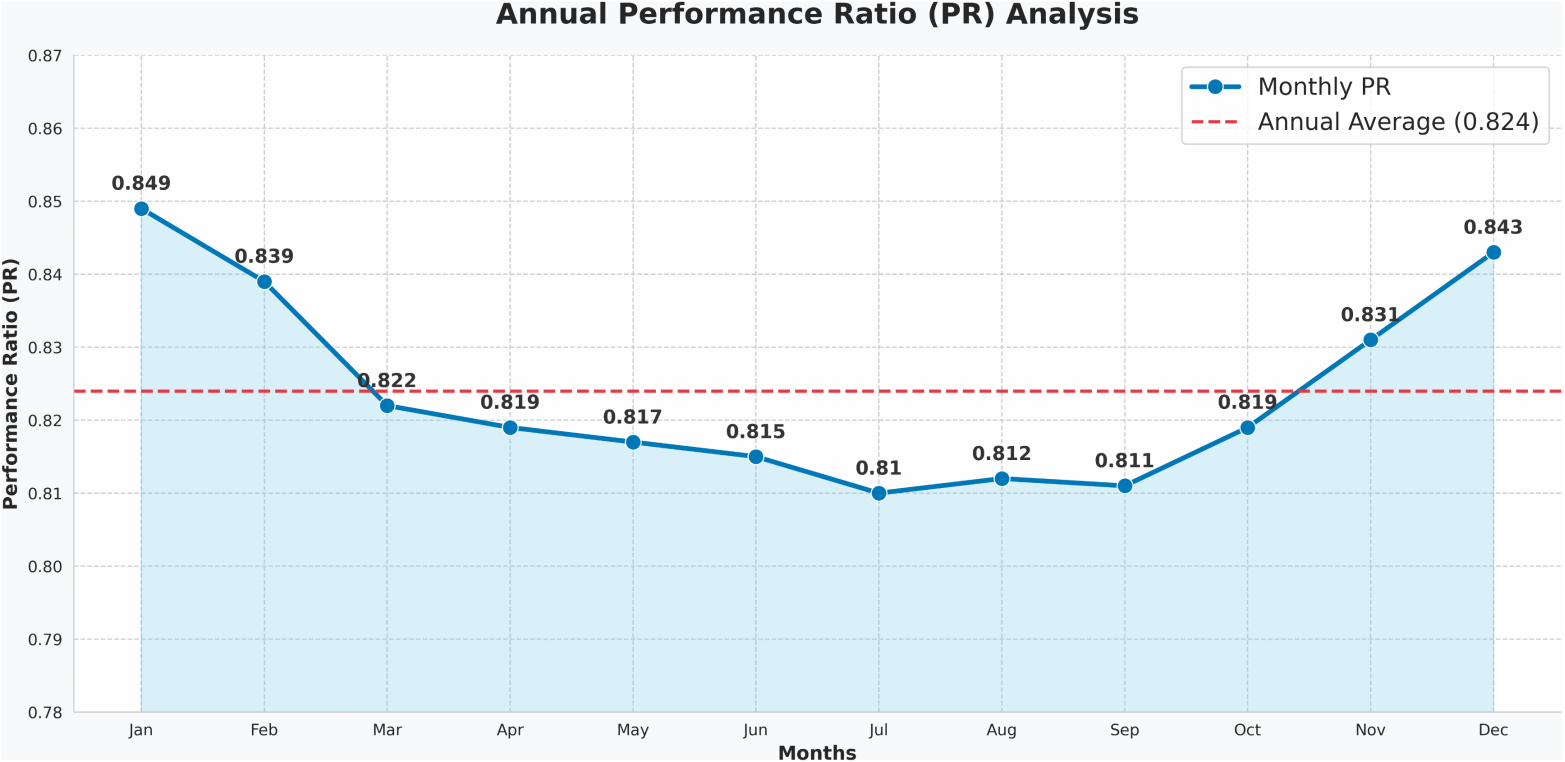
Monthly performance ratio variation of the floating PV system.

The annual performance evaluation of the proposed floating Photovoltaic system was extracted directly from the simulation result reports of the computer program PVsyst, which calculates the time-dependent values of solar resource availability, irradiance after transposition, system losses, and produced electrical energy. The monthly performance indicators of GHI, DHI, EP, effective EP after incidence angle modification, shading EP, EP generated by the array, and the injected-to-grid EP were selected from the simulation results of the computer program PVsyst, as shown in the following Table 3. The sample monthly variation of the Performance Ratio extracted from the computer program simulation results of PVsyst is shown in the Fig 11.

**Table 3:** Monthly solar irradiation and grid-injected energy.

Month	Global horizontal irradiation (kWh/m²)	Horizontal diffuse irradiation (kWh/m²)	Global incident in collector plane (kWh/m²)	Effective global, corrected for IAM and shadings (kWh/m²)	Effective energy at the output of the array (MWh)	Energy injected into the grid (MWh)
**January**	116.6	53.3	136.4	132.7	741.7	729.7
**February**	120.2	62.4	133.2	130.1	716.2	703.9
**March**	160.9	79.0	171.8	168.1	906.8	890.3
**April**	152.6	88.7	155.5	151.5	816.8	802.1
**May**	160.2	102.3	158.5	154.4	830.6	815.5
**June**	130.7	90.9	127.5	123.7	666.7	654.5
**July**	131.8	81.7	129.1	125.6	671.2	658.5
**August**	140.0	90.0	139.8	135.7	728.5	715.3
**September**	124.9	69.6	129.9	126.5	676.7	663.9
**October**	132.7	69.6	144.2	140.5	757.3	743.6
**November**	125.8	47.9	147.3	143.7	785.1	771.4
**December**	115.6	45.7	138.9	135.2	750.3	737.9
**Year**	**1612.0**	**881.5**	**1712.0**	**1667.7**	**9047.9**	**8886.9**

The simulated value of the annual GHI of 1612 kWh/m^2^ acquired from the PVsyst meteorological database proves the high solar resource potential of this location. The high value of the diffuse component (881.5 kWh/m^2^) indicates high humidity with cloud cover, which is typical of wetland sites. However, the effective irradiance calculated by the PVsyst tool of 1667.7 kWh/m^2^ is high, proving that the optical losses associated with the incidence angle, shading, and mismatching were minimized by the chosen array and floating designs. The trends of seasonal energy output reported by PVsyst reveal that the maximum energy output occurs during the pre-monsoon season (March to May) owing to high levels of plane-of-array irradiance. Conversely, the low energy output during the monsoon season (June to September) is largely due to the high module temperatures and the dominance of diffuse irradiance. The difference between the values of the effective energy output from the array and the energy injected into the grid is small and steady, as reported by PVsyst, implying that there are negligible DC-AC conversion losses.

The PR value obtained in a month using PVsyst varied from 0.810 to 0.849, with an average value of 0.824 (82.4%). The higher PR values in winter are due to lower thermal losses and better voltage regulation, whereas the relatively lower values in summer and monsoon are due to temperature-dependent efficiency and mismatch under diffuse irradiation conditions. However, it was observed that the PR variation margin (±2.5%) was very small, which essentially blended the BOS losses and electrical design with minimal sensitivity to seasonal variations and established robustness in the electrical design of the proposed FPV system arrangement. The output analysis of the PVsyst software indicates that the performance of the FPV system is mainly influenced by climatic and thermo-optical conditions over a long period, confirming that the design is sufficiently robust for long-term functioning in tropical wetland environments.

### 4.4 Energy yield normalization and loss breakdown

**Fig 12:**
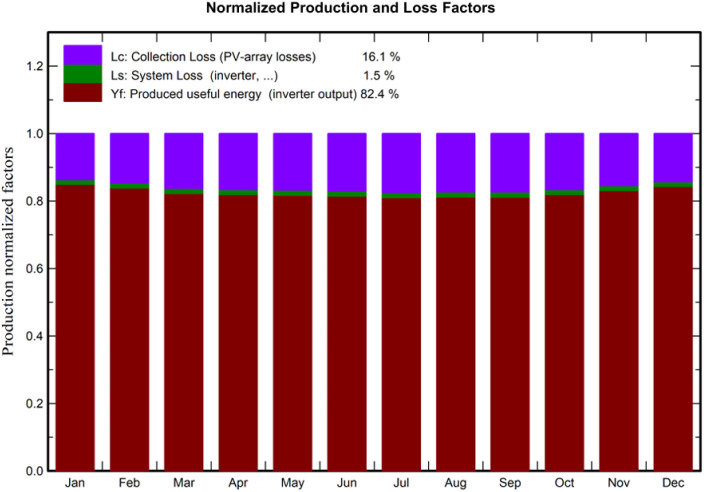
Normalized decomposition of energy yield and system losses.

The performance evaluation of the energy conversion processes of the proposed floating photovoltaic system was performed using the normalized decomposition method for energy yield, which was obtained from the simulation results generated by the PVsyst software. This would make it possible to determine the level of incident solar irradiation that is progressively converted to grid-acceptable electrical energy. It was found that the contributions to energy losses made by the array collection losses account for almost all energy losses, on average, at 16.1%. These are primarily due to optical reflection, angle-of-incidence modifications, and voltage losses under high solar irradiation conditions. The figure (Fig 12) below indicates that the contributions to energy losses made by array collection losses vary somewhat on a seasonal basis, particularly during the summer months, primarily because of the increased temperatures of the PV arrays. However, the losses in the system, which comprise conversion losses and components on the AC side of the inverter, account for only 1.5% of the energy losses per year because of the efficient operation of the selected string inverter type. The narrow variation range for Yf demonstrates that there is good stability between system losses and electrical layout throughout different seasons, which confirms the dominance of FPV technology. The relatively high value of the average performance ratio can also be linked to the thermal moderation that occurs owing to the water mass. Within the simulation environment based on PVsyst, the impact of evaporative cooling occurred indirectly through the temperature operation of floating PV modules, and the low values of module temperature positively influenced the temperature coefficient of the conversion efficiency. Floating PV systems are characterized by less thermal stress than traditional land-based photovoltaics under similar climatic conditions because of evaporative and convective cooling. Thus, losses due to thermal degradation are minimized, leading to increased energy yield stability over time. Nevertheless, it is important to note that the current research does not involve the use of any thermal data obtained directly from the field and through CFD-based thermal modeling for a precise calculation of the heat transfer coefficient associated with evaporation. Therefore, the effect of cooling should be understood in terms of the impact of thermal performance on simulations. In general, the results of the normalized loss analysis revealed that physical factors determine the performance of the energy module.

### 4.5 Long-term performance degradation modeling using climate-aware ML

The long-term operational behavior of the proposed floating photovoltaic (FPV) system was assessed using a climate-informed, machine-learning-assisted degradation framework. Meteorological variables including global horizontal irradiance (GHI), ambient temperature ($T_{amb}$), wind speed (WS), and relative humidity (RH) were extracted from the NASA POWER reanalysis database to represent realistic climatic stressors acting on the system over its operational lifetime. These climatic variables were coupled with system age to jointly capture climate-induced variability and intrinsic aging effects in FPV performance.

The temporal evolution of the performance ratio (PR) was therefore modeled as a degradation process expressed by [9],


$$\begin{array}{c} PR\;(t)=\;{PR}_{o}\;e^{-\beta t}+\varepsilon_{c}(t) \end{array}$$

where $PR_{\text{0}\;}$ is the initial performance ratio obtained from PVsyst simulations, β denotes the effective degradation coefficient learned by the machine-learning model, t is the operational year, and $\varepsilon_{c}(t)\;$ represents stochastic deviations caused by interannual climate fluctuations.

To account for nonlinear interactions between climatic drivers and system aging, a supervised learning model was trained to approximate the functional relationship [44],


$$\begin{array}{c} PR(t)=\;f_{ML}({GHI}_{t},\;T_{amb,\;t},{WS}_{t},\;{RH}_{t},\;t) \end{array}$$

where $f_{ML}(\cdot )\;$ denotes the optimized Random Forest regression model calibrated using PVsyst-generated, physically consistent performance data. This formulation allows the degradation trajectory to adapt dynamically to climate–aging interactions, thereby overcoming the limitations of conventional linear or fixed-rate degradation assumptions. It is emphasized that the ML model functions as a scenario-driven trend estimator, rather than a direct empirical predictor, due to its reliance on simulation-based training data.

The predicted performance ratio was subsequently translated into annual energy output using a physics-consistent scaling approach given by,


$$\begin{array}{c} E(t)=\;E_{\text{1}}\;\frac{PR\;(t)}{{PR}_{o}} \end{array}$$

where $E_{\text{1}}$ is the first-year energy yield obtained from PVsyst. Finally, the cumulative energy degradation over the plant lifetime was quantified as,


$$\begin{array}{c} \Delta E_{cum}(t)=\left(\text{1}-\frac{E(t)}{E_{\text{1}}}\right)\times \text{1}\text{0}\text{0}\% \end{array}$$

**Fig 13:**
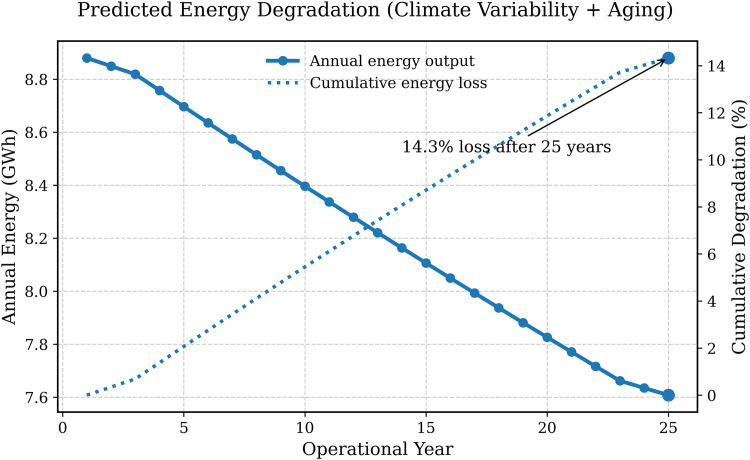
ML-predicted performance ratio degradation under climate variability and aging.

**Fig 14:**
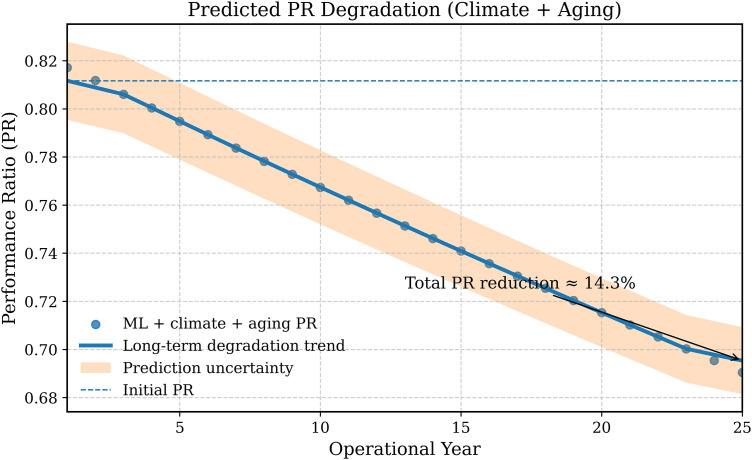
ML-predicted long-term energy degradation and cumulative loss.

The simulated PR degradation and consequent energy loss over a 25-year period are shown in Figs 11 (a)–(b), respectively. The trend lines shown in Figs 13 and 14 are thus a direct result of the predictions obtained by the climate-ML approach and the performance-energy equations. These equations guarantee that the results are entirely mathematically traceable and transparent with respect to the approach used.

### 4.6 Model validation and statistical performance

To further analyze the accuracy and consistency of the proposed climate-informed machine learning model, a thorough validation exercise was conducted on the techno-economic dataset generated by the PVsyst simulator. In training the supervised machine learning model, only the RFR algorithm was used based on its ability to effectively handle medium-sized datasets and identify non-linear relationships among climatic, technical, and economic factors without being overly sensitive to changes in hyperparameters. In this case, the input parameters for the machine learning model were the operational year, annual power production, inflation rate, CAPEX, OPEX, and discount rate, whereas the LCOE was the output variable.

**Fig 15:**
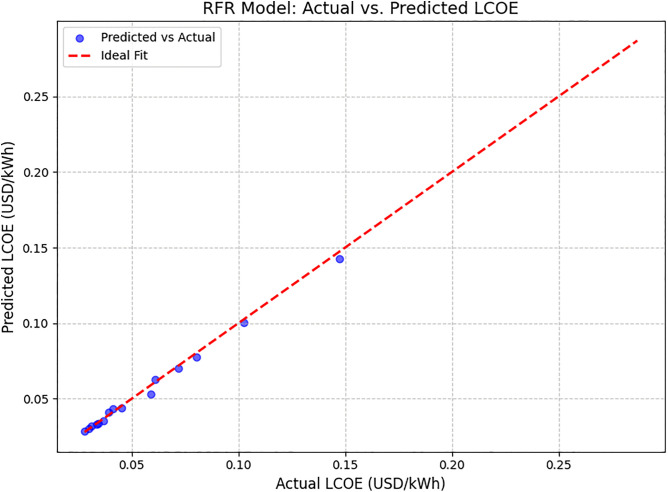
Comparison of actual and random forest regression (RFR)-predicted LCOE values for model validation.

The degradation analysis model considering climate effects is composed of parameters that affect the environmental and operating stresses that impact long-term FPV performance. Of the several parameters used in the study, the annual energy output and the operating cost that is sensitive to inflation had the most impact on the LCOE changes over the lifetime. In contrast, the operating year parameter represents the degradation-sensitive behavior of the system throughout its lifetime. Fig 15 compares the real value of the LCOE and the predicted results using the RFR model. It can be seen that the predicted points are concentrated along the ideal-fit line, indicating a high level of similarity between the predicted value and the real value under different operating conditions.

In addition, the performance of the method was tested using statistical criteria such as the coefficient of determination (R^2^), root mean square error (RMSE), and mean absolute error (MAE). The results obtained from this analysis are summarized in Table 4.

**Table 4:** Statistical validation metrics of the random forest regression (RFR) model for techno-economic LCOE prediction.

Metric	Training	Testing
**R²**	0.942	0.918
**RMSE**	0.0125	0.0158
**MAE**	0.0094	0.0112

The validation statistics show that the formulated RFR model is capable of providing reliable consistency in predicting the LCOE for long-term operation under climate-conscious and inflationary conditions. The minor variation in the values obtained from the training and testing phases indicates that there is little to no overfitting in the prediction process. In addition, the low values of the RMSE and MAE indicate that there is little variance between the predicted output and reference trajectory. The long-term degradation rates generated by the simulation were also in line with the common photovoltaic degradation rate range in the literature, thus confirming the physical validity of the simulation-driven analytical approach presented in this study.

### 4.7 Techno-economic analysis

A techno-economic analysis was performed to determine the financial viability and cost-effectiveness of a proposed floating photovoltaic (FPV) facility with a lifespan of 25 years. The details of the estimated capital and operational expenditures of the project are given in Table 5. The cost estimate considers the import prices of crucial items such as photovoltaic panels, inverters, and transformers, and factors in transport and value-added taxes to ensure a realistic assessment of financial viability at prevailing prices in the local market.

**Table 5:** Capital and operating cost breakdown of the proposed floating photovoltaic (FPV) system [45,46].

Category	Quantity	Cost (USD)
**PV Modules**	9000 pcs	675,000
**Module supports**	9000 sets	90,000
**Inverters**	50 units	175,000
**Accessories & Equipment**		233,000
**Studies & Analysis**		34,000
**Installation & Commissioning**		186,000
**Grid Connection**		120,000
**Plant Preparation**		20,000
**Total Capital Expenditure (CAPEX)**		1,513,000
**Operating & Maintenance (OPEX)**		71,666 USD/year

According to the PVsyst simulations, the proposed FPV system was capable of generating 8.88 GWh per annum at a capacity factor of 20.3%, thus producing approximately 222 GWh of energy during its entire 25-year lifespan. It should be noted that this energy output considers losses at various system levels, such as inverter, mismatching, transformer, and electrical losses due to wiring, among others, which are considered in the PVsyst model itself. But, on the other hand, large-scale curtailments, issues like transmission congestion, and restrictions due to grid dispatches have not been considered in this specific case study. Given that the FPV system proposed here would fall under medium-scale distributed generation facilities, the chance of being subjected to significant curtailments may be lower than that in the current operating environment. The financial assessment is carried out with the assumptions of a feed-in tariff price of 0.10 USD/kWh, a rate of inflation of 5%, and a discount rate of 8% [47]. All CAPEX, OPEX, and LCOE calculations were performed using constant 2024 USD values to maintain consistency across long-term economic projections. For OPEX, which is based on the costs incurred for annual inspection, maintenance, and servicing, the growth rate is assumed to be 8% per year. The depreciable asset value was calculated as 990,000 USD, which considers the behavior of financial matters in the long run. The CAPEX and OPEX figures are based on existing techno-economic assessments for FPV technologies, current market conditions within the region, and cost references of projects published recently. They represent relatively low-cost figures considering the labor and installation expenses in Bangladesh compared to figures reported in the recent literature. With these considerations, the proposed system yields an LCOE of 0.0315 USD/kWh, IRR of 53.17%, and a simple payback period of approximately two years. The comparatively low LCOE is mainly due to the benefits of inland deployment, lack of energy storage requirements, lower balance-of-system costs, and higher energy production because of the bifacial nature of the panels and the water surface albedo effect. As part of the validation process, the calculated LCOE was compared with values available in the literature, wherein FPV systems usually have an LCOE of 0.038–0.065 USD/kWh, whereas ground-mounted photovoltaic systems have an LCOE of 0.10–0.18 USD/kWh. This lower LCOE value was made possible by good site characteristics and an efficient system design, as opposed to impractical assumptions. Moreover, despite conservative estimations that resulted in higher CAPEX or OPEX escalations, the LCOE will still be lower than the average electricity generation cost in Bangladesh (~0.087 USD/kWh).

Moreover, the designed FPV system proves its better cost-effectiveness in terms of the cost of power generation compared to traditional power sources. The calculated LCOE value from the proposed system considerably decreased compared with those of coal-fired power sources (0.15–0.17 USD/kWh), diesel-fired power sources (0.25–0.27 USD/kWh), and traditional ground-mounted solar PV systems (0.10–0.18 USD/kWh) [48,49]. This indicates the better economic viability of the proposed solar PV system with a floating structure. Finally, compared to existing FPV facilities worldwide and one existing FPV facility in the country (Table 6), the system has many advantages in terms of performance and cost-saving efficiencies, in addition to enhanced environment-friendly performance strengths to better apply at Bangladeshi energy markets as well as in several global emerging markets. Overall, the techno-economic performance analysis has been able proved that the proposed FPV system has a unique ability to offer a very low cost of energy, superior economic performance, and comparability to conventional power generation technologies along with existing FPV systems, making it suitable for mega installations in Bangladesh.

**Table 6:** Comparison of the proposed FPV system with existing national and global floating photovoltaic installations [50–54].

FPV Plant Name	Capacity (MWp)	Country	LCOE ($/kWh)
**Huaneng Dezhou Dingzhuang Floating Solar Farm**	320	China	0.065
**NTPC Ramagundam Floating Solar Power Plant**	100	India	0.052
**Malaysia 13 MW Floating Solar (Dengkil)**	13	Malaysia	0.038
**Agongdian Solar PV Park**	10.2	Taiwan	0.060
**Walton Floating Solar Power Plant**	1	Bangladesh	0.040
**Chapainawabganj Floating Solar**	2.3	Bangladesh	0.048
**Proposed FPV plant**	5	Bangladesh	0.0315

### 4.8 Environmental impact and lifecycle carbon assessment

**Fig 16:**
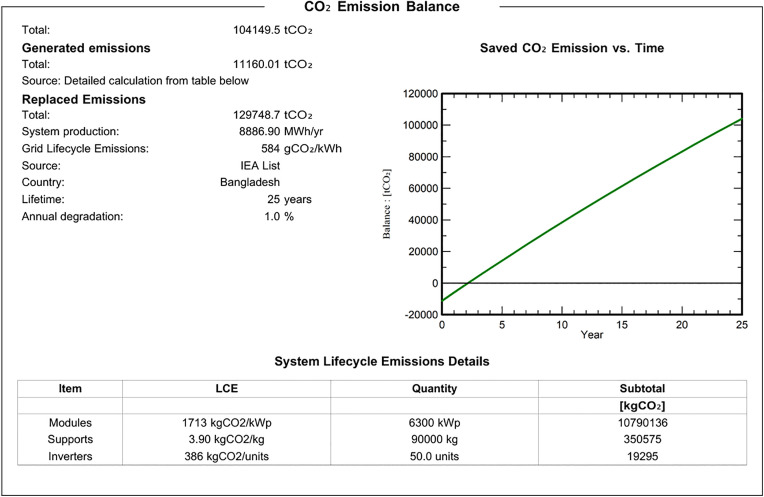
Lifecycle CO₂ balance of the proposed FPV system.

The environmental impact of the FPV system was considered in terms of life cycle CO₂ emissions because there are both emissions caused by the manufacture of the FPV system components and those that are avoided owing to the displacement of electricity generation from power plants. The life cycle CO₂ emissions caused by solar modules, FPV structures, and solar inverters were approximately 11,160.01 tCO₂. However, the FPV system offsets emissions caused by the production of carbon-intensive electricity because of its lifespan of 25 years. With an average annual electricity generation of 8.89 GWh and an average lifecycle emission factor for the Bangladesh grid of 584 gCO2/kWh, the system avoids approximately 129,748.7 tCO2. As shown in Fig 16, to establish the lifecycle CO2 content for the system, an average annual performance ratio degradation rate of approximately 1%, which aligns with the mean trend indicated by the ML performance ratio degradation model, was considered. This led to a net avoidance of approximately 104,149.5 tCO2 after incorporating the embodied CO2 content. It is evident from the existing literature that the emission level of a coal-based power plant is approximately 950 gCO₂/kWh, whereas that of a solar photovoltaic cell is zero [55]. Although the indirect emission level of a PV cell is in the range of 50–60 gCO₂/kWh, the values are well below the existing level of coal-based electricity production. From the analysis, the following results were obtained, and the proposed FPV system emerged as a credible approach for reducing coal-based electricity production in Bangladesh.

## 5 Sensitivity analysis of long-term LCOE

This section introduces the data sources, system of equations, and model employed to assess the long-run economic sensitivity of the proposed floating solar photovoltaic system with respect to operational uncertainties caused by inflation. The proposed model was applied for a 25-year operational life, during which capital costs, operational expenses, and degradation of energy affected the Levelized Cost. The economic dataset used in the analysis was developed using project-specific techno-economic variables, which included the yearly energy output derived from the PVsyst simulation results, initial capital costs (CAPEX), base case operational costs (OPEX), nominal discount rate, and cost paths adjusted to account for inflation. The inflation rate was chosen by adopting internationally acceptable macroeconomic variables as recorded by the World Bank, IMF, and Asian Development Bank, instead of using assumptions. Moderate to high levels of economic stress experienced by countries with emerging markets were reflected in the chosen scenarios of 4%, 8%, and 12% inflation rates.

Operating expenditure is modeled as an inflation-driven compounded process that accounts for salary increments, routine maintenance escalation, spare-part replacement, and auxiliary operational costs. The annual operating cost in year tis expressed as,


$$\begin{array}{c} {OPEX}_{t}=\;{OPEX}_{o}\;.\;(\text{1}+i{)}^{i} \end{array}$$

where ${\text{ OPEX}}_{\text{0}}\;$ denotes the first-year operating cost and i represents the annual inflation rate.

The annual energy output is degradation-adjusted to account for long-term performance decline,


$$\begin{array}{c} E_{t}=\;E_{\text{1}}\;.(\text{1}-d{)}^{t} \end{array}$$

where $E_{\text{1}\;}$ is the first-year energy yield obtained from PVsyst and d   is the annual degradation rate.

The LCOE formulation embedded within the machine-learning framework follows the discounted cash-flow principle [56],


$$\begin{array}{c} LCOE=\;\frac{\sum_{t=\text{1}}^{N}\frac{CAPEX+\;{OPEX}_{t}}{(\text{1}+r{)}^{t}}}{\sum_{t=\text{1}}^{N}\frac{E_{t}}{(\text{1}+r{)}^{t}}} \end{array}$$

where r is the nominal discount rate and N is the project lifetime.

**Fig 17:**
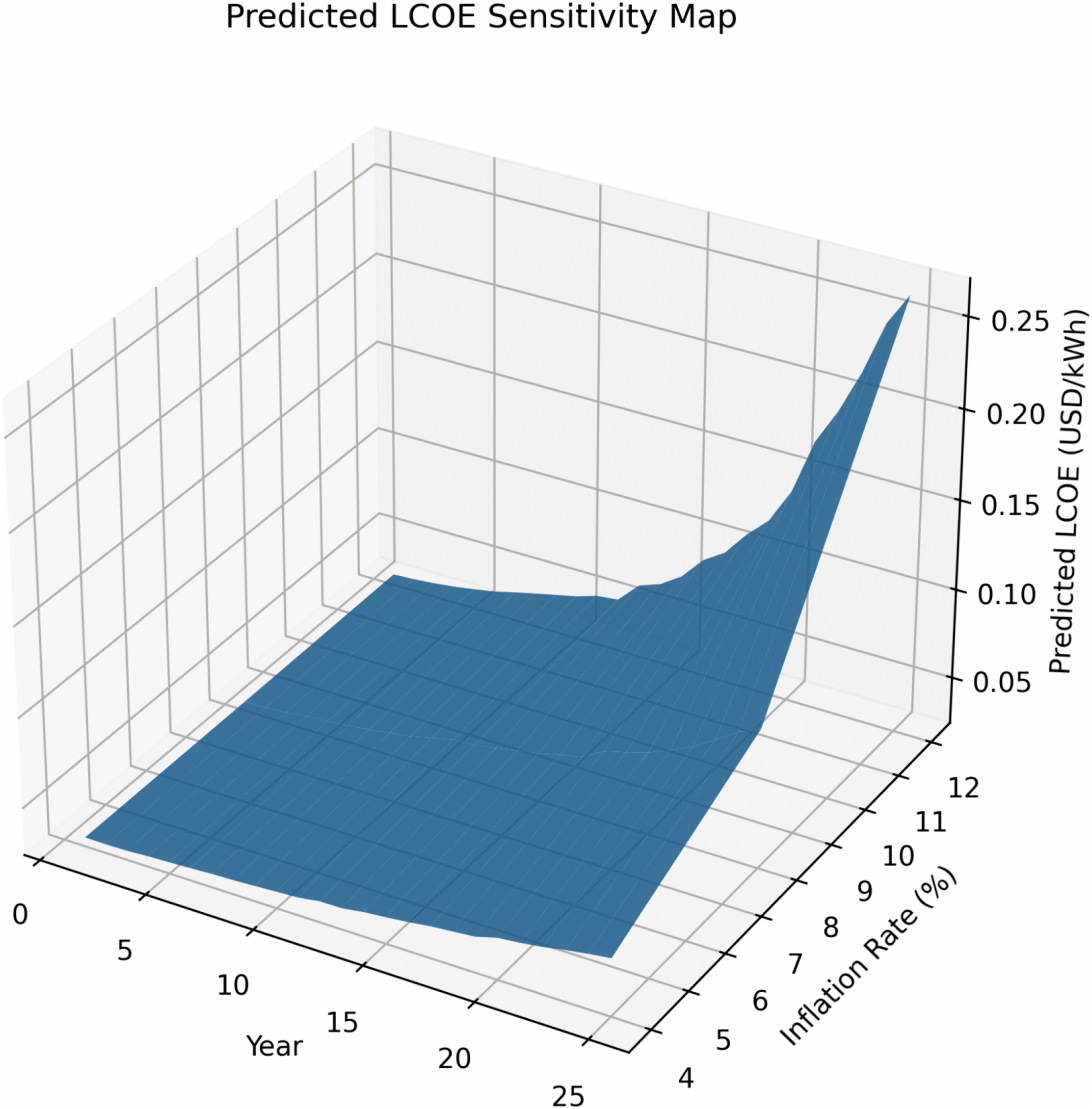
Sensitivity of LCOE to inflation rate and operational lifetime.

Considering time-dependent techno-economic factors, a machine learning prediction framework based on a supervised regression approach was created to evaluate LCOE evolution considering factors such as the operational year, inflation rate, annual power generation, OPEX, discount rate, and CAPEX. The proposed Random Forest Regression model yielded the sensitivity surface shown in Fig 17, wherein variations in the value of LCOE could be represented as a continuous function of the system’s operational life and inflation rate. From the continuous curves obtained for the LCOE in the sensitivity surface, it is evident that the model’s predictions are stable under varying economic conditions. In terms of sensitivity analysis, it is clear that increasing operational costs as a result of inflation exert a progressively greater effect on the evolution of long-term LCOE as opposed to reductions in annual energy generation.

## 6 Discussion

The proposed framework offers an all-encompassing evaluation of an environment-friendly FPV system through a combination of structural, performance, and techno-economic sensitivity analyses, as well as environmental considerations, all within one framework. Based on the findings of this study, environmentally adaptive FPV systems are capable of delivering desirable energy performance levels while at the same time minimizing the land utilization demands of renewable energy projects in areas endowed with abundant water resources. Unlike previous studies on FPV systems that emphasize deterministic energy generation or economic analysis, this study uses machine learning-supported long-term degradation modeling along with inflation-dependent LCOE analysis. In particular, as shown in Table 7, earlier studies used fixed degradation factors, whereas the proposed methodology accounts for the impact of both system degradation and economic variations. The observed sensitivity trends reveal that the change in the LCOE is significantly influenced by the growth in operational expenses, in addition to the degradation processes.

**Table 7:** Comparison of key aspects between past FPV studies and this study.

Aspect	Past Studies	This Study
**FPV structural design**	Conventional HDPE or modular floating platforms [57]	Custom annular eco-permeable pontoon; hydrostatic stability
**Performance modeling**	Static or deterministic yield estimation [58]	PVsyst + ML-based PR and energy degradation
**Economic assessment**	Fixed or simplified LCOE modeling [59]	Inflation-aware, ML-predicted LCOE
**Sensitivity analysis**	Single-parameter sensitivity [60]	3D LCOE sensitivity (inflation–time)
**LCOE dynamics**	Static lifetime-average LCOE [61]	Time-resolved LCOE evolution over 25 years
**Novel contribution**		Integrated pontoon design, ML degradation, and LCOE sensitivity framework.

Structurally, the suggested design of the light-permeable annular pontoon system varies from existing opaque FPVs because it allows PAR radiation to penetrate the water body beneath the pontoons. This innovative design strategy may contribute to minimizing the negative impact of excessive shading effects, which usually characterize opaque FPVs, and consequently enable improved ecological compatibility. Nonetheless, the ecological evaluation provided in this study must be understood as a rudimentary theoretical study based on optical modeling rather than a rigorous ecological validation. The present study did not explicitly consider factors such as oxygen dynamics, thermocline development, nutrient transport, long-term adaptation to biodiversity, and biological measurements in the field. Hence, additional field research is required to determine the ecological implications of the suggested design solution.

Furthermore, the techno-economic evaluation indicates that the system exhibits promising economic viability under the discussed operational scenario. The calculated levelized cost of electricity of 0.0315 USD/kWh is noticeably lower than the current electricity generation cost in Bangladesh, even with conservative inflation assumptions. Moreover, the carbon mitigation potential over the life cycle of approximately 129,748.7 tCO₂ indicates that FPV can make significant contributions to achieving renewable energy transition goals. These results highlight the applicability of FPV systems, considering biodiversity, to national renewable energy strategies, such as the Renewable Energy Policy of Bangladesh and the Mujib Climate Prosperity Plan. Finally, on an international scale, the proposed model is in agreement with the concepts outlined in the IEA Net Zero Roadmap and the Sustainable Development Goals concerning affordable and clean energy and climate action.

Moreover, the modular nature of the designed FPV system, along with the climate-resilient and data-enabled approach to analysis, enhances the suitability of the methodology in other resource-scarce and water-rich regions of South and Southeast Asia. Although the current approach shows great potential for scalability, several real-world issues related to hydrodynamic performance in adverse weather conditions, grid connectivity limitations, siltation, and maintenance over extended periods must be considered in subsequent analyses. In summary, by incorporating the ecological perspective, stability analysis, machine learning-powered degradation modeling, and inflation-based techno-economic analysis, this study established a comprehensive methodology for long-term FPV planning.

## 7 Challenges and future work

Despite the excellent techno-economic and eco-efficiency characteristics of the FPV system, additional studies on some aspects of the design are required. Although hydrostatic stability and buoyancy limitations were tested using theoretical derivations, the dynamics of the system during extreme hydrometeorological events, such as cyclones, storm surges, and monsoon rains, were not specifically considered. Therefore, future FPV plants should be designed with advanced mooring and anchoring mechanisms that can cope with variable water levels and high wind speeds [62]. Regarding the economic aspects of FPV technology, the use of imported bifacial PV modules and converters poses risks associated with exchange rate volatility and tariff implications, which can impact both capital expenditure (CAPEX) and operational expenditure (OPEX)) [63]. Practically, FPV systems have some extra difficulties in maintenance compared to land-based PV systems. Factors such as biofilm formation on PV modules, accumulation of sediments around anchoring systems, and degradation of mooring systems require sophisticated cleaning procedures and maintenance plans [64]. Furthermore, other concerns such as connection to the grid, power quality issues, intermittency problems, and energy storage were not covered by the current research and should be considered in subsequent studies. The implementation of BESS, hydrogen generation technologies, and control strategies (including real-time optimization and adaptive energy management schemes) can contribute to system optimization in the future. In addition, the use of high-fidelity field measurements and the implementation of the concept of digital twins in FPV system assessment is a potential direction.

## 8 Conclusion

This study clearly demonstrates that short-term analysis methods are inadequate for analyzing the long-term feasibility of floating photovoltaic (FPV) plants within inland waters. It has been shown that a more sophisticated evaluation approach is required, including the consideration of the long-term impact of performance degradation and the economic behavior of the system under inflationary conditions. The findings indicate that performance degradation and an increase in operating expenses over time affect the long-term dynamics of the LCOE. This implies that FPVs should not be analyzed under deterministic economic assumptions, as such assumptions do not consider performance degradation and operating costs over time. Considering the environment, the proposed FPV system presents significant opportunities for emissions reductions over its lifecycle compared to the current electricity production process. However, the results of the proposed photosynthetic conservation analysis cannot be considered as a complete ecological validation because the framework only includes a simple optical model and does not consider complex ecosystem interplays such as dissolved oxygen dynamics, thermal stratification, nutrient transfer, biodiversity effects, and aquatic adaptation to new conditions. Therefore, one must apply field-based ecological validation should be applied in future studies. In summary, one can state that the FPV framework in question is applicable in countries with limited space availability but abundant water resources. The application of climate-sensitive PVsyst simulations, techno-economic modeling that considers equipment degradation, and a supervised machine learning approach can help develop a flexible analytical framework for long-term FPV planning. The framework can be further developed by integrating energy storage systems, hydrogen-based energy production pathways, energy management systems, and hydrodynamic reliability studies in extreme weather conditions.

## Supporting information

S1 Fig Graphical abstract. (PNG)

## Abbreviations

FPV: Floating photovoltaic
CAPEX: Capital Expenditure
DEM: Digital Elevation Model
IAM: Incidence Angle Modifier
IRR: Internal Rate of Return
LCOE: Levelized Cost of Electricity
PAR: Photosynthetically Active Radiation
OPEX: Operational Expenditure
MAE: Mean Absolute Error
RFR: Random Forest Regression
ROI: Return on Investment
NASA POWER: Prediction Of Worldwide Energy Resources
O&M: Operation and Maintenance
