# Supplementary figures and images for: Machine learning–based long-term degradation and LCOE Analysis of floating PV with custom pontoon design
Source: PLoS One. 2026 Jul 14;21(7):e0342926. doi: 10.1371/journal.pone.0342926 (PMC13367708; doi:10.1371/journal.pone.0342926)

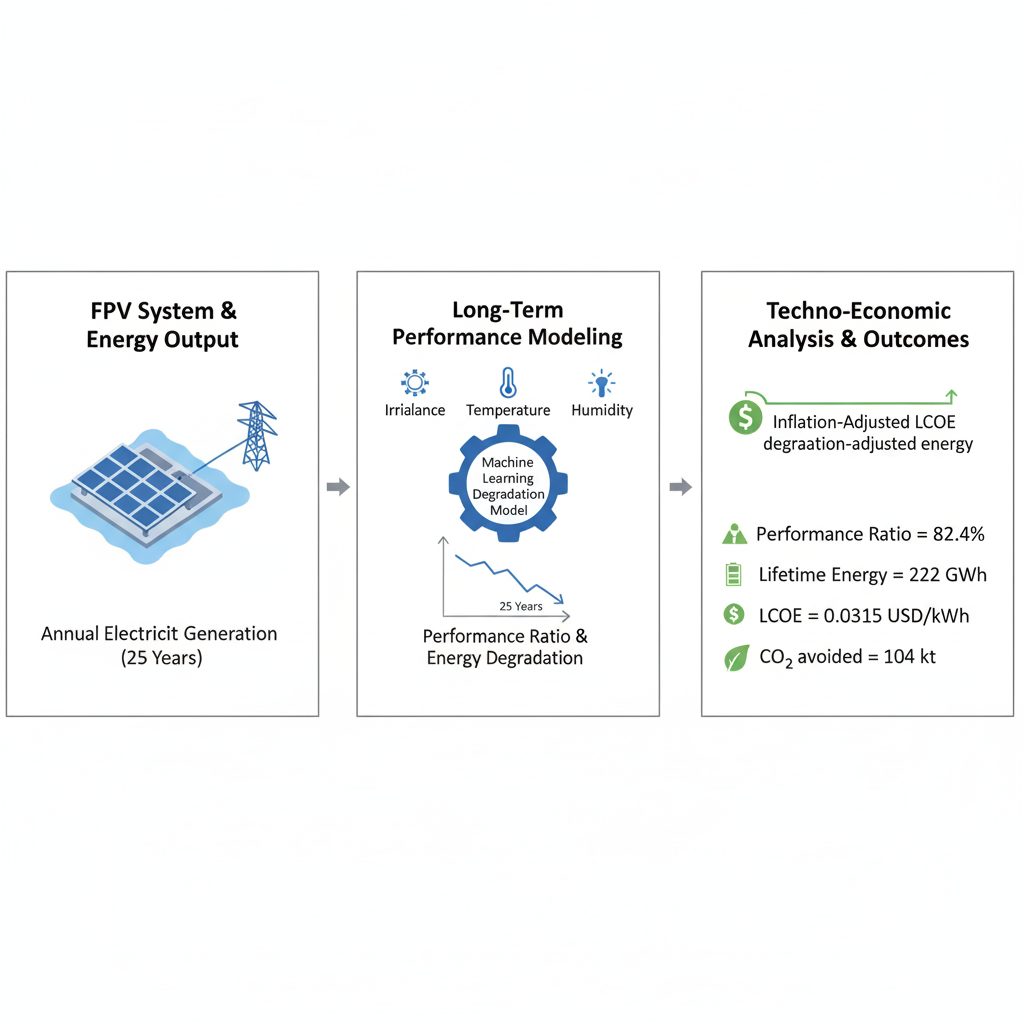

Supplement: S1 Fig — (PNG) [file pone.0342926.s001.png]
